# Effects of argon plasma and aging on the mechanical properties and phase transformation of 3Y-TZP zirconia

**DOI:** 10.1590/0103-6440202204849

**Published:** 2022-10-21

**Authors:** William Matthew Negreiros, Monica Alonso Cotta, Frederick Allen Rueggeberg, Jean Jacques Bonvent, Fabio Dupart Nascimento, Marcelo Giannini

**Affiliations:** 1 Department of Restorative Dentistry, Piracicaba Dental School, University of Campinas, Piracicaba, SP, Brazil.; 2 Department of Applied Physics, Institute of Physics “Gleb Wataghin”, University of Campinas, Campinas, SP, Brazil; 3 Department of Restorative Sciences, Dental College of Georgia, Augusta University, Augusta, GA, USA; 4 Department of Center for Natural and Human Sciences, Federal University of ABC, Santo André, SP, Brazil.; 5 Department of Biochemistry, Paulista School of Medicine, Federal University of São Paulo, São Paulo, SP, Brazil.

**Keywords:** Zirconium, Material Resistance, Phase Transition, Thermal Treatment, State of Matter

## Abstract

To evaluate the flexural strength (FS) and flexural modulus (FM) of a commercial 3Y-TZ0P ceramic after artificial aging and either without or with two application times of non-thermal plasma treatments (NTP). In addition, changes in crystalline phase transformation and surface nano-topography after NTP application, during different aging periods, were evaluated. Ninety 3Y-TZP bars (45x4x3 mm) were made for FS and FM testing, and assigned to nine groups (n=10): no NTP/no aging (Control); no NTP/4h aging; no NTP/30h aging; 10s NTP/no aging; 10s NTP/4h aging; 10s NTP/30h aging; 60s NTP/no aging; 60s NTP/4h aging and 60s NTP/30h aging. Artificial accelerated aging was simulated using an autoclave (134º C at 2 bar) for up to 30h. FS and FM were assessed using a universal testing machine and data analyzed using a ANOVA and Tukey test (α=0.05). The volume change in zirconia monoclinic phase (MPV) was evaluated using X-ray diffraction and surface nano-topography was assessed using atomic force microscopy (baseline until 30h-aging). NTP application did not influence the FS and FM of zirconia. Compared to the Control (no NTP/no aging), the FS of zirconia samples treated for 30 hours in autoclave (“no NTP/30h aging” group) increased. Artificial aging for 30 hours significantly increased the FM of zirconia, regardless of NTP application. MPV tended to increase following the increase in aging time, which might result in the surface irregularities observed at 30h-aging. NTP did not alter the zirconia properties tested, but 30h-aging can change the zirconia FS, FM and MPV.

## Resumo

Avaliar a resistência à flexão (FS) e o módulo de flexão (FM) de uma cerâmica comercial 3Y-TZP após envelhecimento artificial, e com ou sem dois tempos de aplicação de plasma não térmico (NTP). Além disso, a transformação de fase cristalina e a nano-topografia de superfície após a aplicação de NTP, durante diferentes períodos de envelhecimento, foram avaliadas. Noventa barras 3Y-TZP (45x4x3 mm) foram feitas para testes de FS e FM, e distribuídas em nove grupos (n=10): sem NTP/sem envelhecimento (Controle); sem NTP/4h de envelhecimento; sem NTP/30h de envelhecimento; 10sNTP/sem envelhecimento; 10sNTP/4h; 10sNTP/30h; 60sNTP/sem envelhecimento; 60sNTP/4h e 60sNTP/30h. O envelhecimento artificial acelerado foi simulado em autoclave (134º C a 2 bar) por até 30 horas. FS e FM foram avaliados em máquina de ensaio universal e os dados analisados ​​pela ANOVA e teste de Tukey (α = 0,05). A mudança de volume da fase monoclínica de zircônia (MPV) foi avaliada usando difração de raios-X e nano-topografia de superfície foi avaliada utilizando microscopia de força atômica (baseline até 30h). A aplicação do NTP não influenciou a FS e FM da zircônia. Comparado ao Controle (“sem NTP/sem envelhecimento”), a FS das amostras de zircônia tratadas por 30 horas em autoclave (“sem NTP/30h de envelhecimento”) aumentou. O envelhecimento artificial por 30 horas aumentou significativamente a FM da zircônia, independente do tempo de aplicação do NTP. O MPV tendeu a aumentar em função do aumento do tempo de envelhecimento para todos os grupos, que pode ter resultado nas irregularidades superficiais observadas com 30 horas de envelhecimento. O NTP não afetou as propriedades da zircônia testadas, mas o envelhecimento por 30 horas pode alterar a FS, FM e MPV da zircônia.

## Introduction

The understanding of the toughening mechanism of zirconia ceramics in the mid-1970’s contributed to the increase of its biomedical applicability. In Dentistry, the first generation consisted of the 3 mol% yttria-stabilized tetragonal zirconia polycrystalline ceramic (3Y-TZP). It became a prosthetic alternative to porcelain fused-to-metal indirect restorations and castable metal alloy frameworks. The next dental zirconia generations (4Y-TZP and 5-YTZP) have presented superior esthetics and translucency, but with lower mechanical properties ^1^. Considering all Y-TZP generations, clinical applications are wide and range from copings, frameworks, fixed partial dentures, dental implants and monolithic crowns to veneers ^2^. The production of such dental prostheses has been facilitated by CAD/CAM technology ^3^.

Zirconia has three crystallographic forms, depending on temperature. From room temperature to 1170^o^ C, the monoclinic phase (m) predominates. From this temperature to 2370^o^ C, the material takes on a tetragonal phase (t). At temperatures exceeding 2370^o^ C, cubic (c) crystalline phase is present. During cooling, around 950^o^ C, transformation from the “t” to “m” (t→m) phases occurs, and is accompanied by a volumetric increase of approximately 4.5% that compromises the zirconia crystalline structure. In attempt to prevent such phase transformation, zirconia is stabilized with metal oxides (such as Y_2_O_3_) to allow the tetragonal phase to exist in a metastable state at room temperature and hindering t→m transformation ^4^ ^,^ ^5^ ^,^ ^6^.

To achieve durable and strong bond strength of resin cements to zirconia ceramic, their surface must treat by grinding or sandblasting ^7^ ^,^ ^8^ ^,^ ^9^. Also for zirconia implants, it is well-documented that a rough surface is more favorable for osseointegration and endosseous anchorage ^10^. Because the meta-stability of zirconia, stress-inducing treatments having the potential to damage this surface, might promote the t→m transformation. On the one hand, this transformation improves mechanical properties, such as flexural strength, due to formation of compressive stresses that avoid the cracks. However, altering phase integrity may increase the material’s susceptibility to aging, especially for porous surfaces ^11^.

The phenomenon of phase changing near room temperature is referred to as “Low Temperature Degradation” (LTD), and is characterized by the slow, progressive t→m transformation under a wet environment ^12^ ^,^ ^13^. The consequences of such changes have been reported as volumetric changes, micro-crack formation, grain pullout and surface roughening ^2^ ^,^ ^12^ ^,^ ^14^ ^,^ ^15^. To avoid this aging process, one viable option is to alter the surface chemistry, using procedures that are not stress-inducing, and therefore do not result in phase transformation, but still enhance the potential for osseointegration in cases of implants and resin bonding for prosthetic materials.

Among new treatments available for zirconia surface modification is non-thermal plasma (NTP) ^16^ ^,^ ^17^, which has been used for etching, cleaning, decontamination, functionalization, and deposition of films onto the surface of materials ^18^ ^,^ ^19^. This treatment is also a potential mechanism to enhance resin bond strength to zirconia ^16^, and the process works by altering surface chemistry, increasing surface energy and its reactivity, with little deleterious effects compared to other stress-inducing treatments ^20^. The success of this technology is based on the application of high doses of active species at room temperature that chemically destabilizes surfaces ^18^.

No investigation has been performed to test the effect of NTP on t→m transformation of a commercial dental zirconia. If deleterious transformations do not occur, then the NTP may be better accepted as a surface-activating treatment for enhancing resin bonding to an activated zirconia ceramic surface. Thus, the purpose of the present study was to evaluate the effect of artificial aging and NTP treatment on the flexural strength, flexural modulus, crystalline phase transformation and surface nano-topography of a commercial 3Y-TZP ceramic. The research hypotheses were that: 1- the NTP and 2- the hydrothermal accelerated artificial aging do not affect the flexural strength, modulus and t→m zirconia transformation of a 3Y-TZP zirconia.

## Materials and methods

A commercially available, 3Y-TZP dental zirconia block (IPS e.max ZirCAD; Ivoclar Vivadent, Schaan, Leichtenstein) was used to investigate the effects of NTP and artificial aging in autoclave. Ninety bar-shaped specimens were cut using a precision cutter (Isomet 1000; Buehler Ltd, Lake Bluff, IL, USA), into predetermined dimensions to compensate for sintering shrinkage, and to meet ISO 13356 standards (final dimensions of specimens were: 45 mm in length; 4.0 +/- 0.2 mm in width and 3.0 +/- 0.2 mm in thickness). Specimens were polished using 600 and 1200 grit abrasive papers, and finally using cloth discs combined with 3 (m and 1 (m diamond polishing pastes (MetaDi; Buehler Ltd, Lake Bluff, IL, USA). Subsequently, specimens were sintered at 1450^o^ C for 1 h, to prevent an increase in cubic content ^6^. Thermal annealing was performed for 20 min at 1350^o^ C, to expose grain boundaries and to ensure only the formation of tetragonal grains. Specimens were randomly assigned into nine groups (n = 10, eight experimental groups and the Control), as shown in Table 1.

**Table 1:** Experimental groups and abbreviations, according to the argon plasma application and artificial aging times.

Group abbreviation	NTP application	Artificial aging
NTPNA	none	none
NTP4A	none	4 hours
NTP30A	none	30 hours
10NTPNA	10 seconds	none
10NTP4A	10 seconds	4 hours
10NTP30A	10 seconds	30 hours
60NTPNA	60 seconds	none
60NTP4A	60 seconds	4 hours
60NTP30A	60 seconds	30 hours

The NTP equipment used (Model SAP; Surface - Engineering and Plasma Solution Ltd, Campinas, SP, Brazil) consisted of a hand-held unit (130 mm length x 30 mm diameter), with a quartz nozzle (4 mm length x 2 mm diameter) attached to a high voltage power supply used to produce a non-thermal plasma torch at atmospheric pressure. High purity argon (Praxair 4.8; White Martins, Rio de Janeiro, RJ, Brazil) was used at 5 liters per minute to produce a plasma torch. The cold thermal “torch” exiting the nozzle was 20 mm long x 2 mm diameter and was operated at room temperature of 22^o^ C. The hand-held unit was fixed in a lab support stand and the distance between plasma-jet nozzle end and the zirconia surface was 10 mm, with the hand-held unit placed vertically. One side of zirconia bar (45 mm in length and 4.0 mm in width) received each one 10 or 60 seconds of NTP and in order to treat it homogeneously, NTP was consecutively applied to nine areas (each 5 mm) of this specimen side. 

The hydrothermal accelerated artificial aging of sixty zirconia bars was performed in an autoclave (Vitale Plus; Cristófoli, Campo Mourão, PR, Brazil) for 4 or 30 hours (with steam at 134^o^C, under two bar pressure). Thirty zirconia samples were not aged in autoclave and used as baseline.

To determine flexural strength and flexural modulus of the zirconia samples treated or not with NTP and artificial aging, bar-shaped specimens underwent a four-point bending test, as described by ISO 13356. Flexural strength was measured using 4-point (20 x 40 mm) spans, with the loading applied to the opposite surface that was treated with NTP. Testing was conducted at ambient temperature, on a universal testing machine (model 4411; Instron Corp., Canton, MA, USA), at a crosshead speed of 1 mm/minute to avoid slow crack growth before failure. Collected data consisted of the maximum load until failure and modulus of both aged and non-aged (control) groups. Flexural strength (MPa) and flexural modulus (GPa) data were obtained using a software (Bluehill 2; Instron Corp., Canton, MA, USA), checked for normal distribution using Shapiro-Wilk test and were each analyzed using a two-way ANOVA (“Aging” and “Treatment” factors) followed by the Tukey post-hoc test. All statistical analysis was performed using a pre-set alpha of 0.05. 

Before the flexural strength test, one specimen from the groups “NTP30A”, “10NTP30A”, “60NTP30A” was subjected to x-ray diffraction (XRD) analysis, using CuKα radiation (40 kV, 40 mA) (D8 Advanced; Bruker, Karlsruhe, Germany). The XRD analysis were evaluated after different periods of artificial aging (0, 2, 4, 6, 8, 10, 15, 20, 30 hours), so that change in the volume of the monolithic phase could be investigated. All scans were performed using an angulation range of 27-33 degrees (2ϴ) at a scan speed of 0.2 degrees/minute, with a step size of 0.02 degrees.

The monoclinic phase fraction (*X*_m_) was measured using the Garvie and Nicholson formula ^36^:

$$X_{m}\;=\;\frac{I_{m}(-111)\;+\;I_{m}(111)}{I_{m}(-111)\;+\;I_{m}(111)\;+\;I_{t}t(101)}$$ (1)

Where I_t_ and I_m_ represent the integrated intensity (area under peaks) of the tetragonal (101) and monoclinic (111) and (-111) peaks. Monoclinic volume content was calculated as described by Toraya et al:^37^

$$V_{m}=\frac{1.311X_{m}}{1\;+\;0.311X_{m}}$$ (2)

Also, three specimens from all groups, including those used in XRD analysis were evaluated for changes in surface nano-topography, using atomic force microscopy (Model SPM 9600; Shimadzu Corp., Kyoto, Japan). Topography images of the specimens were obtained with contact mode using 10-μm scan size.

## Results

Flexural strengths and flexural moduli for experimental groups and the Control are described in Tables 2 and 3, respectively. The two-way ANOVA and Tukey test demonstrated that NTP did not significantly influence either of these test parameters within each of the artificial aging times used (0, 4, and 30 hours). For the NTP30A group (no NTP application), the aging time for 30 hours significantly increased in flexural strength, compared to the baseline condition (p < 0.05) (Table 2). For each NTP-treatment group, flexural moduli were significantly greater after 30 h of artificial aging than the baseline (no aging) and after 4 h of aging (p < 0.05) (Table 3). No interaction between “Aging” and “Treatment” was found (p > 0.05) for either test parameter: flexural strength or flexural modulus.

**Table 2:** Flexural strength means (SD) of 3Y-TZP (in MPa), according to the argon plasma application and the artificial aging time.

Treatment		Artificial Aging (Time)	
	baseline	4 hours	30 hours
none	358 (50) B a	495 (155) AB a	556 (189) A a
10NTP	402 (98) A a	412 (175) A a	488 (148) A a
60NTP	483 (115) A a	393 (120) A a	557 (213) A a

Upper case letter compare aging times for the same treatment (row) and lower case letters compare treatments for the same artificial aging time (column) (n = 10 specimens/group). NTP: Non-thermal plasma.

**Table 3:** Flexural modulus means (SD) of 3Y-TZP (in GPa), according to the argon plasma application and the artificial aging time.

Treatment		Artificial Aging (Time)	
	baseline	4 hours	30 hours
none	180 (23) B a	165 (26) B a	236 (28) A a
10NTP	170 (17) B a	164 (21) B a	237 (26) A a
60NTP	170 (18) B a	171 (28) B a	237 (25) A a

Upper case letter compare aging times for the same NTP treatment (row) and lower case letters compare treatments for the same artificial aging time (column) (n = 10 specimens/group). NTP: Non-thermal plasma.

**Figure 1:**
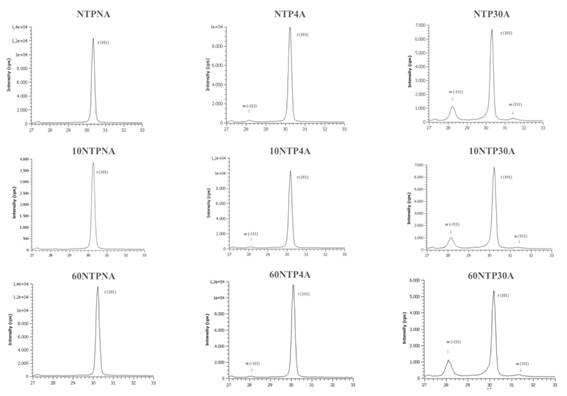
X-ray diffraction patterns (at the range of 27-33 degrees, 2ϴ) of 3Y-TZP specimens treated with argon plasma and artificial aging. Monoclinic (m); Tetragonal (t). m-phase peaks (111 and -111) were observed from 4 hours of aging and increased the intensity at 30 hours.

**Figure 2:**
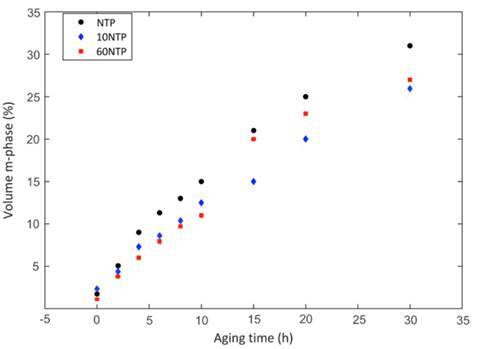
Volume of monoclinic phase (%) as a function of artificial aging time (hours) of 3Y-TZP specimens treated with argon plasma (10NTP and 60NTP) or not (NTP). The curves show the increase in monoclinic phase transformation with aging for all groups.

**Figure 3:**
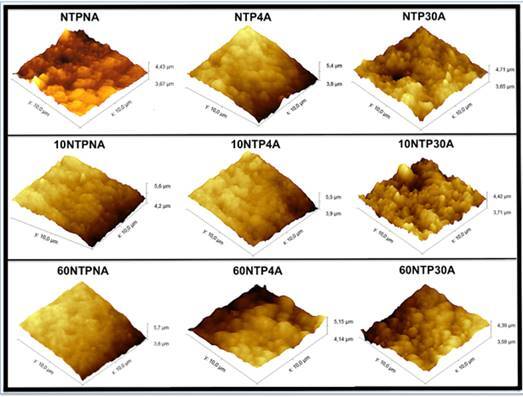
AFM topography images (10 μm scan size) showing the nano-topography of the 3Y-TZP specimens treated with argon plasma and artificial aging

The XRD analysis (Figure 1) shows no presence of m-phase transition for the baseline (no artificial aging). At 4 hours of aging, the first signs of t→m transformation were detected. For the sample subjected to 30 hours of aging, a noticeable increase in intensity of the -111 and 111 peaks are seen, indicating the influence of long-term artificial aging on the t→m transformation. Changes in the monoclinic phase volume (Figure 2) showed that all groups presented t→m transformation after aging, and that the increase appears lower for groups treated with NTP, although these values were not submitted to statistical analysis. 3Y-TZP nano-topography images showed that the surface irregularities tended to increase at 30h of aging, regardless of the NTP application (Figure 3).

## Discussion

The first research hypothesis stating that NTP would not affect the flexural strength, flexural modulus and not accelerate t→m zirconia transformation of 3Y-TZP was accepted, while the second one stating that artificial aging would not affect such properties was rejected. When flexural strengths and moduli were analyzed, an increase in both values after 30 hours of accelerated aging was observed (Tables 2 and 3). However, flexural strength statistically increased only for NTP30A group. The increase in both tests could be explained by the higher m-phase content present in the zirconia (Figures 1 and 2), suggesting that a t→m transition promotes the development of compressive pressure that increases the load required to fracture the sample. ^4^. However, this increase in compressive pressure might is indicative of internal stress that can have negative consequences such as the catastrophic failure of the zirconia structure ^15^.

Other studies have also shown increased in the mechanical properties of Y-TZP ceramics and reported less than 50% of m-phase volume as resulted of similar autoclave aging ^14^ ^,^ ^21^. In this study, the increasing of m-phase volume after 30 hour of artificial aging was also observed and the values of m-phase volume were: 31.2% for NTP30A, 26.0% for 10NTP30A, and 27.0% for 60NTP30A. 

The ISO 13356 instructions were followed to prepare the zirconia samples and according to the International Standard, the dental zirconia should contain less than 20% of m-phase to be clinically accepted. In this study, the threshold value was reached after 20 hours of artificial aging for 10NTP experimental group and after 15 hours for NTP and 60NTP groups. At 30 hours of aging, the m-phase volume appeared to be higher when 3Y-TZP zirconia was not treated with NTP (Figure 2). 

Some theories have explained the mechanism of LTD of Y-TZP zirconia ^6^ ^,^ ^14^ ^,^ ^10^ ^,^ ^13^ ^,^ ^22^ and the most accepted model is that the moisture (H_2_O), when in contact with the ZrO_2_ ceramic, reacts with O_2_-forming hydroxyl groups (OH^-^). Subsequently, these hydroxyl groups permeate within grain boundaries, bond to oxygen vacancies present in the Y-TZP, and form proton structural defects, destabilizing the tetragonal phase. The t→m transformation is classified as a “martensitic transformation”, where there is a change in structural coordination and change in structure of crystals, without atom diffusion ^5^.

A study suggested that one-hour of autoclave artificial aging (at 134^o^ C and two bars pressure) represents 2-3 years of in vivo environmental exposure ^22^. In this study, zirconia samples were aging in autoclave for four and thirty hours, that represented 8-12 and 60-90 years, respectively. The artificial aging of the zirconia samples using autoclave has been used to analyze the t→m transformation at 37^o^ C in a moist environment ^2^ ^,^ ^4^ ^,^ ^10^ ^,^ ^12^ ^,^ ^13^ ^,^ ^15^. 

To identify the effects of the NTP application on the mechanical properties of 3Y-TZP zirconia, this study aged the specimens immediately after treating the samples with argon plasma, because surfaces treated with NTP are reactive for up to 5 hours ^17^. Authors suggested that the hydrophilic potential of NTP lasts for only a short period; therefore, the specimens must be aged as fast as possible. Phase volume analysis showed that NTP treated surfaces did not induce accentuated t→m transformation (Figure 2). The argon plasma application tended to slightly reduce the t→m transformation after 30 hours of aging, partially protecting the 3Y-TZP against the hydrothermal aging, although these values were not submitted to statistical analysis.

XRD analysis did not detect m-phase peaks (111 and -111) before aging (at baseline), even for those groups treated with argon plasma. At 4 hours of aging, the first signs of zirconia phase transformation with increasing of m-phase content were detected, regardless of NTP application. For the sample subjected to 30 hours of aging, a noticeable increase in intensity of the -111 and 111 peaks are seen, indicating the strong influence of artificial aging by autoclave on the t→m transformation (Figure 1). The first and second generations of zirconia ceramics (3Y-TZP) have showed tetragonal to monoclinic phase transformation after 100 days in the oral environment with increased roughness and changes in mechanical properties ^23^. The intensity of this t→m transformation can be related to the surface irregularities observed for the zirconia samples subjected to 30 hours of aging (Figure 3).

In the study of Hallmann et al. ^24^, Y-TZP specimens were abraded with different air-borne particles (alumina and silica-coated alumina) and the acceleration aging of specimens was performed under autoclave (134^o^C and a water vapor pressure of 2.3 bar) for 2 hours. The results indicated that the roughness of YTZP zirconia surface increased after accelerated artificial aging. Another study showed detachment of grains from the surface of two commercial 3Y-TZP zirconia ceramics associated with volume increase of grains after 24-hours aging by autoclave ^21^, which increased the zirconia surface roughness ^9^ ^,^ ^12^ ^,^ ^24^.

Because some studies have reported concerns about the longevity of resin cement adhesion to zirconia ^7^ ^,^ ^8^, the application of argon plasma to zirconia in order to improve adhesion ^25^ and reduce the t→m transformation effects during the intra-oral aging could keep the zirconia restoration in good clinical condition for a long time. In this study, it was not possible to obtain the zirconia surface roughness data using atomic force microscopy to accurately discuss whether autoclave aging changes the nano-topography of the zirconia surface. Only one specimen per group was used in XRD analysis, because of the long evaluation time of each sample and the high cost of using the XRD equipment. Another limitation of this study was the artificial aging in autoclave and the autoclaving times that stipulate years of the zirconia natural aging in the human oral cavity. 

The results of this study suggested that: 1- Non-thermal plasma treatment of 3Y-TZP did not affect either flexural strength or flexural modulus and did not induce the higher t→m transformation; 2- Artificial aging in an autoclave for up to 30 hours can increase the flexural modulus and the monoclinic phase content, independent on argon plasma application, and 3- Argon plasma may reduce the t→m transformation and keep stable the flexural strength of a 3Y-TZP zirconia after artificial aging for 30 hours.
